# Chronic Diseases and Sociodemographic Characteristics Associated With Online Health Information Seeking and Using Social Networking Sites: Nationally Representative Cross-sectional Survey in Japan

**DOI:** 10.2196/44741

**Published:** 2023-03-02

**Authors:** Seigo Mitsutake, Yoshimitsu Takahashi, Aki Otsuki, Jun Umezawa, Akiko Yaguchi-Saito, Junko Saito, Maiko Fujimori, Taichi Shimazu, INFORM Study Group

**Affiliations:** 1 Human Care Research Team Tokyo Metropolitan Institute for Geriatrics and Gerontology Tokyo Japan; 2 Department of Health Informatics Kyoto University School of Public Health Kyoto Japan; 3 Division of Behavioral Sciences National Cancer Center Institute for Cancer Control National Cancer Center Tokyo Japan; 4 Division of Prevention National Cancer Center Institute for Cancer Control National Cancer Center Tokyo Japan; 5 Division of Cohort Research National Cancer Center Institute for Cancer Control National Cancer Center Tokyo Japan; 6 Faculty of Human Sciences Tokiwa University Ibaraki Japan; 7 Division of Supportive Care, Survivorship and Translational Research National Cancer Center Institute for Cancer Control National Cancer Center Tokyo Japan; 8 See Acknowledgments

**Keywords:** chronic diseases, cross-sectional study, eHealth literacy, health communication, internet, social networking

## Abstract

**Background:**

In an aging society, worsening chronic diseases increase the burden on patients and the health care system. Using online health information including health information via social networking sites (SNSs), such as Facebook and YouTube, may play an important role in the self-management of chronic diseases and health promotion for internet users.

**Objective:**

This study aims to improve strategies for promoting access to reliable information for the self-management of chronic diseases via the internet, and to identify populations facing barriers to using the internet for health, we examined chronic diseases and characteristics associated with online health information seeking and the use of SNSs.

**Methods:**

This study used data from the INFORM Study 2020, which was a nationally representative cross-sectional postal mail survey conducted using a self-administered questionnaire in 2020. The dependent variables were online health information seeking and SNS use. Online health information seeking was assessed using 1 question about whether respondents used the internet to find health or medical information. SNS use was assessed by inquiring about the following 4 aspects: visiting SNSs, sharing health information on SNSs, writing in an online diary or blog, and watching a health-related video on YouTube. The independent variables were 8 chronic diseases. Other independent variables were sex, age, education status, work, marital status, household income, health literacy, and self-reported health status. We conducted a multivariable logistic regression model adjusted for all independent variables to examine the associations of chronic diseases and other variables with online health information seeking and SNS use.

**Results:**

The final sample for analysis comprised 2481 internet users. Hypertension or high blood pressure, chronic lung diseases, depression or anxiety disorder, and cancer were reported by 24.5%, 10.1%, 7.7%, and 7.2% of respondents, respectively. The odds ratio of online health information seeking among respondents with cancer was 2.19 (95% CI 1.47-3.27) compared with that among those without cancer, and the odds ratio among those with depression or anxiety disorder was 2.27 (95% CI 1.46-3.53) compared with that among those without. Further, the odds ratio for watching a health-related YouTube video among those with chronic lung diseases was 1.42 (95% CI 1.05-1.93) compared with that among those without these diseases. Women, younger age, higher level of education, and high health literacy were positively associated with online health information seeking and SNS use.

**Conclusions:**

For patients with cancer, strategies for promoting access to websites with reliable cancer-related information as well as access among patients with chronic lung diseases to YouTube videos providing reliable information may be beneficial for the management of these diseases. Moreover, it is important to improve the online environment to encourage men, older adults, internet users with lower education levels, and those with low health literacy to access online health information.

## Introduction

As the global population ages, the development of strategies for improving health is becoming increasingly critical. Chronic diseases become more prevalent with aging, causing an increasing burden on patients and the health care system [1,2]. In Japan, a previous study reported that over 90% of adults aged 75 or older have 1 chronic disease, and, of those, approximately 80% have multiple chronic diseases [1]. It is thus important to optimize health care strategies to support people with chronic diseases.

The internet has become widely used as a tool for improving individuals’ health-related knowledge and behavior. National surveys in the United States and Japan reported that over 80% of the general population had used the internet [3,4]. Recently, internet use has increased among older adults in developed countries [4,5]. In a previous study, approximately 80% of patients with chronic diseases reported having searched for health information using the internet [6]. Recent reviews reported that health information seeking on the internet can improve physician-patient relationships [7,8]. The internet has therefore become an important information tool for health promotion, and is widely used in health care settings. More recent development of internet-based applications has provided methods for interactive health-related communication using social networking sites (SNSs) such as Facebook and Twitter. SNSs are widely used and varied, and provide opportunities for health-related communication to support individuals with chronic diseases [4,9,10]. Identifying chronic diseases associated with health information seeking and the use of SNSs may help to improve the strategies used to promote access to reliable information for self-management of chronic diseases via the internet. However, only few studies have examined the associations between different chronic diseases and online health information seeking and SNS use.

Several prior studies have examined the characteristics associated with online health information seeking and SNS use [10-14]. Two studies reported that individuals who seek health information on the internet were more likely to be younger, women, non-Hispanic white, and to have higher socioeconomic status [11,12]. Furthermore, younger adults were reported to be more likely to use SNSs for health communication [10], while younger adults and those with higher levels of education were found to be more likely to watch health-related videos on YouTube [13]. Although these prior studies were conducted in the United States, few studies have also examined the associations of sociodemographic factors with online health information seeking and SNS use in Asian populations. It is thus important to identify the characteristics associated with online health information seeking and various aspects of SNS use to identify target populations that need assistance in seeking health information via the internet and SNSs.

In this study, we identified chronic diseases associated with online health information seeking and the use of SNSs to improve strategies for promoting access to reliable information for the self-management of chronic diseases via the internet. In addition, to identify populations facing barriers to using the internet for health purposes, we examined characteristics associated with online health information seeking and the use of SNSs.

## Methods

### Study Design and Participants

We conducted a nationally representative cross-sectional postal mail survey using a self-administered questionnaire (INFORM Study 2020). Questionnaire items were initially selected from the Health Information National Trends Survey (HINTS) [15]. The INFORM Study 2020 was designed to monitor information relevant to cancer (eg, cancer awareness, knowledge, attitudes) nationally, and to identify the populations most in need of cancer-related information. The sampling method of the INFORM Study 2020 was based on the census conducted by the Japanese government. A total of 10,000 Japanese individuals were sampled using a 2-stage stratified random sampling method, with census area as the primary sampling unit and individuals aged 20 years or older as the secondary sampling unit. From 35 strata crossing 9 regions and 4 municipality groups by population size, we randomly selected 500 census areas with a probability that was proportional to the size of the stratum. We set the total sample size at 10,000 to retain the margin of error of the recommended value (0.05) from the World Health Organization (WHO) sample size calculator using a 35% response rate to the questionnaire, on the basis of the HINTS [16,17]. A more detailed description of this sampling method was reported in a prior study [18].

We mailed the invitation letter and the questionnaire to 10,000 individuals. Of these, 281 were undelivered. Data collection for the INFORM Study 2020 began on August 1, 2020, and concluded on September 30, 2020. We excluded participants that did not use the internet, as assessed by question “Do you ever go online to access the internet or World Wide Web, or to send and receive email?” in the INFORM Study 2020 questionnaire, because our analysis focused only on participants who had used the internet [18]. Participants who had even 1 missing data point, or provided no answers in relation to the dependent variables or independent variables without the income variable, were also excluded.

### Ethics Approval

The INFORM Study 2020 protocol was approved by the Research Ethics Committee of the National Cancer Center (research project number 2019-290) and the Ethics Committee of the Tokyo Metropolitan Geriatric Hospital and Institute of Gerontology (research project number: 2020-32). We considered the participants who selected the item “Agree to participate” in the introductory statement of the questionnaire as consenting to participate in the study.

### Measures

#### Online Health Information Seeking and SNS Use

The dependent variables were online health information seeking and SNS use. Online health information seeking was assessed by the following question in the INFORM questionnaire: “In the past 12 months, have you used the internet to look for health or medical information for yourself?” Participants answered either “yes” or “no.”

SNS use was assessed using 4 subquestions after the following context was provided: “Sometimes people use the internet to connect with other people online through social networks like Facebook, Twitter, Instagram, and LINE. This is often called *social media.*” The 4 subquestions were as follows: “In the last 12 months, have you used the internet for any of the following reasons?: (1) to visit an SNS, such as Facebook; (2) to share health information on SNS, such as Facebook or Twitter; (3) to write in an online diary or blog (ie, web log); and (4) to watch a health-related video on YouTube. The participants answered either “yes” or “no,” and participants who answered “yes” were categorized as using SNSs in each of the 4 questions. We did not use the data for 1 subquestion of “to participate in an online forum or support group for people with a similar health or medical issue” because very few participants answered “yes” to conduct multivariable analyses (n=33).

#### Chronic Diseases

The independent variables of 8 chronic diseases were assessed using the question of “Have you ever been diagnosed as having cancer?” and 7 subquestions of “Has a doctor ever told you had any of the following medical conditions?” The 7 subquestions asked about the following conditions: (1) diabetes or high blood sugar; (2) hypertension or high blood pressure; (3) heart diseases such as heart attack, angina, or congestive heart failure; (4) cerebrovascular diseases including stroke; (5) chronic lung diseases such as asthma, emphysema, or chronic bronchitis; (6) arthritis or rheumatism; (7) depression or anxiety disorder. The participants answered either “yes” or “no.”

#### Sociodemographic Variables and Other Variables

We included sex, age, education status, work, marital status, household income, health literacy, and self-reported health status as the sociodemographic variables and other variables in our analyses. Education status was divided into 3 categories (≤high school graduate, career college and junior college, and ≥college graduate). Regarding work status, participants who answered that they had a job were categorized as “yes,” and participants who answered “none” or “students” were categorized as “no.” Regarding marital status, participants who answered “married” were categorized as “married,” and participants who answered “never married,” “widowed,” or “divorced” were categorized as “not married.” Furthermore, participants were sorted into 6 categories according to annual household income (“<2 million yen,” “2 to <4 million yen,” “4 to <6 million yen,” “6 to <8 million yen,” “8 to <10 million yen,” and “≥10 million yen”; US $1=JPY 105.9) [19]. We measured health literacy using a tool that included 5 questions to assess communicative and critical health literacy [20]. Participants answered using a 5-point Likert scale, ranging from 1 (strongly disagree) to 5 (strongly agree) for whether they could do the following: (1) obtain health-related information from various sources; (2) extract the required information; (3) understand and communicate the information obtained; (4) assess the reliability of the information; and (5) make decisions based on the information, specifically in the context of health-related issues. The scores for the 5 questions were summed and divided into 4 categories using quartile of the scores (5-15, 16-18, 19-20, and ≥21). Furthermore, participants reported their subjective health status as “excellent,” ”very good,” “good,” ”fair,” or “poor”: they were then classified as either “excellent-good” or “fair-poor.”

### Statistical Analysis

First, the chi-square test was used to compare the proportion of online health information seeking and SNS use among patients with various characteristics. We then examined the associations of chronic diseases and other variables with online health information seeking and SNS use using a multivariable logistic regression model that was adjusted for all variables (sex, age, education status, work, marital status, household income, health literacy, self-reported health status, and 8 chronic diseases). Given the results of the national survey, we weighted data by multiplying the reciprocal of the probability of selecting the participants for the survey by the reciprocal of the probability of nonresponse with reference to the HINTS methodology [21]. The probability of nonresponse was estimated based on strata, sex, and age information according to our survey sampling method, which differs from the HINTS. We conducted a weighted analysis to account for the complex sampling design and missing responses to calculate accurate population parameter estimates and 95% CIs for the Japanese general population using the Taylor series linearization method [22]. All analyses were conducted using SPSS version 28.0 (IBM Corp).

## Results

### Study Participant Selection

**Figure 1:**
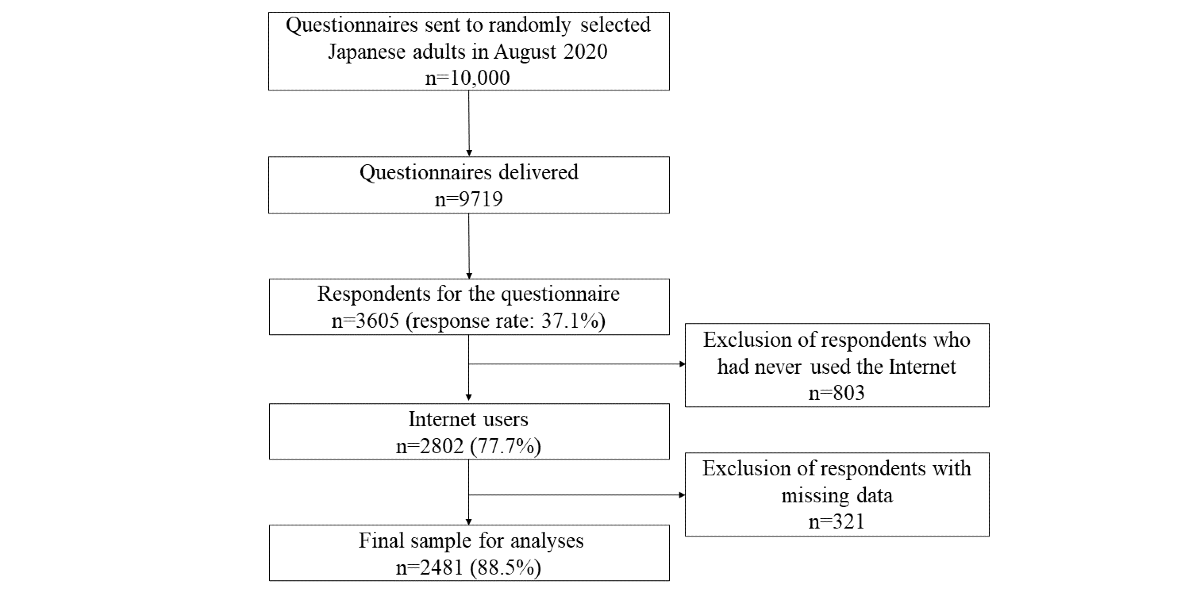
Flow chart of patient selection in the INFORM Study 2020.

Figure 1 shows the participant selection process. A total of 3605/9719 responses were obtained (response rate=37.1%). We excluded 803 respondents who had never used the internet, and included 2802 respondents who had used the internet (77.72%). In addition, we excluded 321 respondents with missing data. The final sample for analysis comprised 2481 participants.

### Characteristics of Study Participants

Table 1 summarizes the weighted sample characteristics of internet users. Approximately 51.6% of internet users were men, and 24.1% were 40-49 years old. Approximately 24.5% of internet users had high blood pressure or hypertension, 10.1% had chronic lung diseases, 7.7% had depression or anxiety disorder, and 7.2% had cancer. In addition, approximately 72.9% of internet users had sought health information and 64.1% of internet users had used SNSs. Regarding SNS use, 55.3% of internet users had visited an SNS, 12.9% had shared health information on an SNS, 26.6% had watched a health-related video on YouTube, and 6.6% had written in an online diary or blog.

**Table 1:** Weighted sample of internet users (n=2481).

Characteristics			Weighted percentage^a^
**Sex**			
	Men	51.6	
	Women	48.4	
**Age groups (years)**			
	20-29	14.3	
	30-39	17.3	
	40-49	24.1	
	50-59	19.4	
	60-69	14.4	
	≥70	10.5	
**Education status**			
	≤High school graduate	37.5	
	Career college/junior college	24.3	
	≥College graduate	38.2	
**Work**			
	No	25.8	
	Yes	74.2	
**Marital status**			
	Not married	32.3	
	Married	67.7	
**Household income (million yen^b^)**			
	<2	5.6	
	2 to <4	21.6	
	4 to <6	23.8	
	6 to <8	19.5	
	8 to <10	12.4	
	≥10	14.9	
	No answer	2.1	
**Health literacy**			
	5-15	19.6	
	16-18	30.7	
	19-20	34.6	
	≥21	15.1	
**Self-reported health status**			
	Excellent, very-good, good	79.9	
	Fair-poor	20.1	
**Chronic diseases^c^**			
	Cancer	7.2	
	Diabetes or high blood sugar	6.9	
	Hypertension or high blood pressure	24.5	
	Heart diseases	3.9	
	Cerebrovascular diseases including stroke	1.0	
	Chronic lung diseases	10.1	
	Arthritis or rheumatism	3.8	
	Depression or anxiety disorder	7.7	
**Dependent variables^c^**			
	Online health information seeking	72.9	
**SNSs^d^ use^c^**			
	Visiting SNSs	55.3	
	Sharing health information on SNSs	12.9	
	Watching a health-related video on YouTube	26.6	
	Writing in an online diary or blog	6.6	

^a^Results were weighted to be representative of the population of internet users in Japan.

^b^US $1=JPY 105.9.

^c^Includes only those who answered “yes.”

^d^SNS: social networking site.

### Comparisons of Chronic Diseases and Other Variables and Online Health Information Seeking and SNS Use

Table 2 presents the comparisons of characteristics of online health information seeking among internet users. A higher proportion of internet users with depression or anxiety disorder sought health information on the internet compared with those without these diseases. Higher proportions of women than men, those who were employed compared with those who were unemployed, and those who experienced worse health status were more likely to seek health information on the internet. In addition, internet users who were 30-39 years old, those with the highest level of education, those with the highest household income, and those with the highest health literacy level exhibited the highest proportions of online health information seeking.

**Table 2:** Comparisons of characteristics of online health information seeking.

Characteristics				Weighted percentage^a^		*P* value^b^		
**Chronic conditions**								
	**Cancer**							
		No	72.4		.05			
		Yes	79.1					
	**Diabetes or high blood sugar**							
		No	73.1		.43			
		Yes	70.4					
	**Hypertension or high blood pressure**							
		No	73.6		.15			
		Yes	70.6					
	**Heart diseases**							
		No	72.8		.70			
		Yes	74.5					
	**Cerebrovascular diseases including stroke**							
		No	59.3		.18			
		Yes	72.9					
	**Chronic lung diseases**							
		No	72.4		.13			
		Yes	77.0					
	**Arthritis or rheumatism**							
		No	72.7		.37			
		Yes	77.0					
	**Depression or anxiety disorder**							
		No	71.8		<.001			
		Yes	86.5					
**Sociodemographic variables and other variables**								
	**Sex**							
		Men	69.9		.001			
		Women	76.2					
	**Age groups (years)**							
		20-29	75.9		<.001			
		30-39	77.9					
		40-49	75.3					
		50-59	72.9					
		60-69	70.6					
		≥70	58.2					
	**Education status**							
		≤High school graduate	66.7		<.001			
		Career college/junior college	75.6					
		≥College graduate	77.3					
	**Work**							
		No	68.1		.003			
		Yes	74.6					
	**Marital status**							
		Not married	71.8		.45			
		Married	73.4					
	**Household income (million yen^c^)**							
		<2	69.3		<.001			
		2 to <4	66.6					
		4 to <6	74.6					
		6 to <8	76.3					
		8 to <10	73.9					
		≥10	77.9					
		No answer	56.3					
	**Health literacy**							
		5-15	61.5		<.001			
		16-18	73.3					
		19-20	74.7					
		≥21	82.8					
	**Self-reported health status**							
		Excellent, very-good, good	71.7		.008			
		Fair-poor	77.7					

^a^Results were weighted to be representative of the population of internet users in Japan.

^b^Chi-square test.

^c^US $1=JPY 105.9.

Table 3 shows the comparisons of the characteristics of SNS users. A higher proportion of internet users without cancer reported visiting an SNS compared with those with cancer. A higher proportion of internet users with chronic lung diseases reported watching a health-related video on YouTube compared with those without chronic lung diseases. A higher proportion of internet users with depression or anxiety disorder reported using all domains of SNS compared with those without these diseases. Moreover, women, younger adults, and those with the highest level of education exhibited the highest proportions of visiting an SNS and writing in an online diary or blog. Internet users that were employed were more likely to visit an SNS and share health information on an SNS. Higher proportions of those who were married compared with those who were not married, and those who had the highest level of health literacy compared with those with the lowest level of health literacy used all domains of SNS. In addition, higher proportions of those with the highest household income and those who experienced worse health status visited an SNS.

**Table 3:** Comparisons of characteristics of SNS^a^ use.

Characteristics				Visiting an SNS					Sharing health information on SNSs					Watching a health-related video on YouTube					Writing in an online diary or blog			
				%^b^		*P* value^c^		%^b^			*P* value^c^		%^b^			*P* value^c^		%^b^				*P* value^c^
**Chronic conditions**																						
	**Cancer**																					
		No	56.9		<.001		13.2			.12		27.0			.16		6.8				.08	
		Yes	35.1				9.0					22.2					3.5					
	**Diabetes or high blood sugar**																					
		No	56.7		<.001		13.1			.24		27.2			.03		6.7				.51	
		Yes	35.8				9.7					19.3					5.3					
	**Hypertension or high blood pressure**																					
		No	60.8		<.001		14.3			<.001		27.7			.03		7.4				.005	
		Yes	38.2				8.5					23.2					4.0					
	**Heart diseases**																					
		No	56.1		<.001		13.1			.07		26.9			.15		6.6				.55	
		Yes	34.9				6.8					20.4					5.1					
	**Cerebrovascular diseases including stroke**																					
		No	46.9		.42		11.3			.81		23.2			.69		3.2				.44	
		Yes	55.3				12.9					26.6					6.6					
	**Chronic lung diseases**																					
		No	55.5		.57		12.6			.23		25.9			.02		6.4				.38	
		Yes	53.5				15.4					32.9					7.9					
	**Arthritis or rheumatism**																					
		No	55.9		.001		12.9			.88		26.6			.77		6.4				.03	
		Yes	38.2				12.3					27.9					11.9					
	**Depression or anxiety disorder**																					
		No	54.3		.002		12.2			.001		26.1			.03		6.2				.01	
		Yes	67.0				20.8					33.3					11.3					
**Sociodemographic and other variables**																						
	**Sex**																					
		Men	52.8		.01		12.7			.79		25.1			.10		5.5				.03	
		Women	57.9				13.1					28.2					7.7					
	**Age groups (years)**																					
		20-29	85.1		<.001		24.5			<.001		38.4			<.001		10.2				<.001	
		30-39	75.6				19.1					34.1					9.6					
		40-49	61.6				13.9					23.4					7.9					
		50-59	45.4				8.2					22.4					4.0					
		60-69	30.9				5.0					23.7					4.1					
		≥70	18.4				3.7					17.7					1.8					
	**Education status**																					
		≤High school graduate	46.4		<.001		12.0			.48		25.7			.74		5.8				.04	
		Career college/junior college	57.3				12.6					27.5					5.3					
		≥College graduate	62.8				13.9					27.0					8.2					
	**Work**																					
		No	45.5		<.001		10.1			.02		26.6			.99		6.3				.73	
		Yes	58.7				13.8					26.6					6.7					
	**Marital status**																					
		Not married	62.2		<.001		16.8			<.001		30.1			.01		8.3				.02	
		Married	51.9				11.0					25.0					5.7					
	**Household income (million yen^d^)**																					
		<2	43.9		<.001		11.6			.27		27.0			.26		10.1				.31	
		2 to <4	44.2				9.9					24.9					5.6					
		4 to <6	55.2				13.7					29.2					6.6					
		6 to <8	59.7				13.7					27.4					7.8					
		8 to <10	60.2				12.7					29.4					5.9					
		≥10	64.6				14.6					22.2					5.1					
		No answer	64.8				19.7					22.2					9.7					
	**Health literacy**																					
		5-15	48.5		<.001		10.9			<.001		24.2			.19		5.0				<.001	
		16-18	55.9				10.7					25.2					6.9					
		19-20	54.8				12.5					28.0					5.1					
		≥21	64.0				20.6					29.6					11.3					
	**Self-reported health status**																					
		Excellent, very-good, good	57.6		<.001		13.3			.24		26.5			.76		6.9				.17	
		Fair-poor	46.0				11.3					27.2					5.1					

^a^SNS: social networking site.

^b^Results were weighted to be representative of the adult population of internet users in Japan.

^c^Chi-square test.

^d^US $1=JPY 105.9.

### Associations of Chronic Diseases and Characteristics With Online Health Information Seeking and SNS Use

Table 4 shows the associations of chronic diseases and characteristics with online health information seeking adjusted for all variables. The odds ratio of online health information seeking among those with cancer was 2.19 (95% CI 1.47-3.27) compared with that among those without cancer, and the odds ratio among those with depression or anxiety disorder was 2.27 (95% CI 1.46-3.53) compared with that among those without depression or anxiety disorder. In addition, compared with men, women were more likely to seek health information on the internet, as were adults aged 20-29 years compared with adults aged 40 years or older, college graduates compared with high school graduates or lower, those who were married compared with those who were not married, those with health literacy scores of 16 or higher compared with those with health literacy scores of 5-15, and those who experienced worse health status compared with those who experienced good health status.

**Table 4:** Association of characteristics with online health information seeking.

Characteristics			Online health information seeking, adjusted odds ratio (95% CI^a^)
**Chronic conditions**			
	**Cancer**		
		No	Reference
		Yes	2.19 (1.47-3.27)
	**Diabetes or high blood sugar**		
		No	Reference
		Yes	1.07 (0.73-1.56)
	**Hypertension or high blood pressure**		
		No	Reference
		Yes	1.10 (0.87-1.38)
	**Heart condition**		
		No	Reference
		Yes	1.47 (0.92-2.35)
	**Cerebrovascular diseases including stroke**		
		No	Reference
		Yes	0.53 (0.19-1.51)
	**Chronic lung disease**		
		No	Reference
		Yes	1.20 (0.88-1.65)
	**Arthritis or rheumatism**		
		No	Reference
		Yes	1.30 (0.78-2.17)
	**Depression or anxiety disorder**		
		No	Reference
		Yes	2.27 (1.46-3.53)
**Sociodemographic variables and other variables**			
	**Sex**		
		Men	Reference
		Women	1.56 (1.25-1.94)
	**Age groups (years)**		
		20-29	Reference
		30-39	0.90 (0.59-1.37)
		40-49	0.67 (0.46-0.98)
		50-59	0.57 (0.38-0.85)
		60-69	0.52 (0.35-0.78)
		≥70	0.32 (0.20-0.50)
	**Education status**		
		≤High school graduate	Reference
		Career college/junior college	1.24 (0.96-1.60)
		≥College graduate	1.55 (1.23-1.95)
	**Work**		
		No	Reference
		Yes	1.18 (0.91-1.53)
	**Marital status**		
		Not married	Reference
		Married	1.27 (1.00-1.61)
	**Household income (million yen^b^)**		
		<2	Reference
		2 to <4	0.77 (0.47-1.25)
		4 to <6	1.02 (0.62-1.67)
		6 to <8	1.01 (0.60-1.70)
		8 to <10	0.86 (0.50-1.48)
		≥10	1.01 (0.59-1.74)
		No answer	0.40 (0.19-0.86)
	**Health literacy**		
		5-15	Reference
		16-18	1.82 (1.38-2.41)
		19-20	1.97 (1.52-2.57)
		≥21	3.12 (2.17-4.50)
	**Self-reported health status**		
		Excellent, very-good, good	Reference
		Fair-poor	1.63 (1.26-2.12)

^a^Multivariable logistic regression model adjusted for all variables.

^b^US $1=JPY 105.9.

Table 5 shows the associations of chronic diseases and characteristics with SNS use adjusted for all variables. The odds ratio for watching a health-related video on YouTube among those with chronic lung diseases was 1.42 (95% CI 1.05-1.93) compared with that among those without chronic lung diseases. In addition, the odds ratios for visiting an SNS and sharing health information on an SNS among participants with depression or anxiety disorder were 1.49 (95% CI 1.04-2.15) and 1.67 (95% CI 1.13-2.46) compared with those among participants without depression or anxiety, respectively. Moreover, women and internet users with higher levels of education were more likely to visit an SNS and write in an online diary or blog. Younger adults and those with higher health literacy were more likely to use all domains of SNS. Participants whose annual income was over 10 million yen were more likely to visit an SNS than those whose annual income was lower than 2 million yen, and less likely to write in an online diary or blog.

**Table 5:** Associations of characteristics and chronic diseases with SNS^a^ use.

Characteristics				Visiting an SNS, AOR^b^ (95% CI)^c^		Sharing health information on SNSs, AOR (95% CI)^c^		Watching a health-related video on YouTube, AOR (95% CI)^c^		Writing in an online diary or blog, AOR (95% CI)^c^			
**Chronic conditions**													
	**Cancer**												
		No	Reference		Reference		Reference		Reference				
		Yes	0.93 (0.64-1.34)		1.35 (0.76-2.42)		0.99 (0.67-1.46)		0.84 (0.35-2.02)				
	**Diabetes or high blood sugar**												
		No	Reference		Reference		Reference		Reference				
		Yes	1.02 (0.70-1.48)		1.31 (0.71-2.42)		0.78 (0.51-1.19)		1.32 (0.61-2.83)				
	**High blood pressure or hypertension**												
		No	Reference		Reference		Reference		Reference				
		Yes	0.83 (0.67-1.02)		1.01 (0.70-1.45)		1.06 (0.83-1.35)		0.89 (0.54-1.49)				
	**Heart condition**												
		No	Reference		Reference		Reference		Reference				
		Yes	0.98 (0.64-1.48)		0.66 (0.29-1.50)		0.80 (0.47-1.38)		0.99 (0.34-2.92)				
	**Cerebrovascular diseases including stroke**												
		No	Reference		Reference		Reference		Reference				
		Yes	1.81 (0.79-4.18)		1.36 (0.31-5.96)		0.97 (0.38-2.47)		0.53 (0.03-9.85)				
	**Chronic lung disease**												
		No	Reference		Reference		Reference		Reference				
		Yes	1.00 (0.72-1.38)		1.21 (0.80-1.82)		1.42 (1.05-1.93)		1.20 (0.69-2.07)				
	**Arthritis or rheumatism**												
		No	Reference		Reference		Reference		Reference				
		Yes	0.90 (0.55-1.46)		1.50 (0.72-3.13)		1.16 (0.72-1.85)		3.19 (1.53-6.64)				
	**Depression or anxiety disorder**												
		No	Reference		Reference		Reference		Reference				
		Yes	1.49 (1.04-2.15)		1.67 (1.13-2.46)		1.27 (0.90-1.78)		1.62 (0.98-2.68)				
**Sociodemographic variables and other variables**													
	**Sex**												
		Men	Reference		Reference		Reference		Reference				
		Women	1.32 (1.07-1.63)		0.99 (0.75-1.32)		1.14 (0.92-1.41)		1.52 (1.07-2.17)				
	**Age groups (years)**												
		20-29	Reference		Reference		Reference		Reference				
		30-39	0.51 (0.32–0.83)		0.81 (0.52–1.25)		0.83 (0.58–1.21)		1.09 (0.61–1.94)				
		40-49	0.25 (0.16-0.39)		0.52 (0.35-0.79)		0.48 (0.33-0.71)		0.92 (0.52-1.65)				
		50-59	0.13 (0.08-0.21)		0.27 (0.17-0.44)		0.46 (0.31-0.67)		0.45 (0.23-0.89)				
		60-69	0.08 (0.05-0.12)		0.17 (0.09-0.31)		0.48 (0.31-0.72)		0.42 (0.21-0.87)				
		≥70	0.05 (0.03-0.08)		0.13 (0.06-0.27)		0.32 (0.20-0.52)		0.17 (0.06-0.50)				
	**Education status**												
		≤High school graduate	Reference		Reference		Reference		Reference				
		Career college/junior college	1.15 (0.90-1.47)		0.92 (0.64-1.33)		1.02 (0.79-1.32)		0.77 (0.48-1.21)				
		≥College graduate	1.59 (1.24-2.02)		0.96 (0.69-1.33)		1.03 (0.80-1.33)		1.51 (1.00-2.29)				
	**Work**												
		No	Reference		Reference		Reference		Reference				
		Yes	0.98 (0.78-1.23)		1.13 (0.79-1.61)		0.91 (0.71-1.15)		0.92 (0.58-1.47)				
	**Marital status**												
		Not married	Reference		Reference		Reference		Reference				
		Married	1.10 (0.87-1.40)		0.89 (0.65-1.21)		1.00 (0.78-1.28)		0.89 (0.60-1.33)				
	**Household income (million yen^d^)**												
		<2	Reference		Reference		Reference		Reference				
		2 to <4	1.17 (0.78-1.77)		0.96 (0.50-1.83)		0.98 (0.61-1.55)		0.53 (0.24-1.18)				
		4 to <6	1.48 (0.97-2.26)		1.23 (0.63-2.41)		1.15 (0.73-1.83)		0.54 (0.24-1.19)				
		6 to <8	1.49 (0.97-2.27)		1.14 (0.58-2.26)		1.02 (0.63-1.66)		0.60 (0.26-1.35)				
		8 to <10	1.52 (0.95-2.43)		1.06 (0.53-2.13)		1.16 (0.68-1.98)		0.44 (0.18-1.08)				
		≥10	1.79 (1.12-2.86)		1.22 (0.60-2.48)		0.77 (0.46-1.31)		0.35 (0.15-0.83)				
		No answer	1.72 (0.81-3.63)		1.59 (0.58-4.41)		0.67 (0.31-1.48)		0.67 (0.21-2.14)				
	**Health literacy**												
		5-15	Reference		Reference		Reference		Reference				
		16-18	1.50 (1.14-1.98)		1.05 (0.71-1.54)		1.14 (0.86-1.49)		1.52 (0.93-2.49)				
		19-20	1.64 (1.25-2.17)		1.41 (0.97-2.05)		1.38 (1.04-1.81)		1.17 (0.69-1.98)				
		≥21	1.99 (1.43-2.77)		2.31 (1.52-3.50)		1.41 (1.03-1.94)		2.52 (1.47-4.30)				
	**Self-reported health status**												
		Excellent, very-good, good	Reference		Reference		Reference		Reference				
		Fair-poor	0.88 (0.69-1.11)		0.97 (0.70-1.34)		1.17 (0.90-1.52)		0.70 (0.44-1.10)				

^a^SNS: social networking site.

^b^AOR: adjusted odds ratio.

^c^Multivariable logistic regression model adjusted for all variables.

^d^US $1=JPY 105.9.

## Discussion

### Principal Findings

This national representative cross-sectional survey identified chronic diseases and characteristics associated with online health information seeking and SNS use among Japanese internet users. Internet users with cancer and those with depression/anxiety disorder were more likely to seek health information on the internet than those without these diseases. Those with chronic lung diseases were more likely to watch a health-related video on YouTube than those without chronic lung diseases, and those with depression or anxiety disorder were more likely to visit an SNS and share health information on SNS than those without depression or anxiety disorder. In addition, women, younger age, higher education status, being married, having higher health literacy, and experiencing worse health status were predictive variables associated with online health information seeking. Moreover, women and higher education status were predictive of visiting an SNS and writing in an online diary or blog. Younger adults and those with higher health literacy were also more likely to use all domains of SNS.

Internet users with cancer were more likely to seek health information on the internet than those without cancer. This result is in accordance with the findings of a previous national cohort study in the United States [23]. Approximately 80% of internet users with cancer sought health information on the internet in this study, which was consistent with data from the HINTS [3]. Several studies reported that online health information seeking among patients with cancer has increased in recent years [24,25]. Access to appropriate information regarding cancer can contribute to improving anxiety and the quality of life (QOL) among patients with cancer [26,27]. Therefore, online health information seeking plays an increasingly important role in clinical settings, and in the behavior and QOL of patients with cancer. However, many websites contain unreliable and difficult to understand cancer information [28-31]. Misinformation can negatively affect health behavior and patient-physician relationships [7,32]. Therefore, an effective strategy may be for medical staff in hospitals to specify websites containing reliable cancer-related information to patients with cancer. Moreover, eHealth literacy, defined as the ability to seek, find, understand, and appraise health information on the internet to address or resolve a health problem, may be important for using health information on the internet effectively [33-35]. Therefore, it is important to improve the strategy for increasing eHealth literacy among internet users with cancer. Although a recent prior study reported effective eHealth literacy interventions for older adults using both text and pictures, such as illustrations and animation [36], only few studies have examined the effect of eHealth literacy interventions while adjusting for confounders such as education level. Therefore, it is necessary to examine the effect of eHealth literacy interventions while considering the influence of confounders.

In this study, compared with internet users without chronic lung diseases, the odds of watching a health-related video on YouTube among those with chronic lung diseases were 1.4 times higher. This study is the first to show that internet users with chronic lung diseases are more likely to watch a health-related video on YouTube. Education is important for people with chronic lung diseases because self-management skills such as problem solving, decision making, or resource utilization for patients with chronic lung diseases are associated with their QOL [37,38]. Several previous studies indicated that YouTube videos can be useful educational tools for asthma or chronic obstructive pulmonary disease [39,40]. The results of our study indicated that publishing the video content of asthma or chronic obstructive pulmonary disease on YouTube may provide an effective education strategy for patients with chronic lung diseases. For example, the Japan Asthma Society has published several videos about how to use inhaled drug treatment on YouTube [41]. However, several prior studies indicated that YouTube contains a substantial amount of low-quality information about chronic lung disease [39,40]. Therefore, strategies to help patients with chronic lung diseases and low levels of eHealth literacy to access high-quality videos via YouTube, and to improve patients’ eHealth literacy, are important.

The results of this study indicated that internet users with depression or anxiety disorder were more likely to seek health information on the internet, and to visit and share health information on SNS compared with those without these diseases. These results are consistent with those of several studies showing a positive association between the prevalence of mental conditions and online health information seeking [42,43]. These associations may be affected by cyberchondria, which is defined as increased anxiety caused by excessive online health information seeking and has recently become a common problem [43]. However, a prior study showed a negative association between depression and online health information seeking among older women [25]. In particular, patients with severe depression are less likely to seek health information on the internet owing to a loss of interest [44]. Thus, only few individuals with severe depression would have participated in this study because many individuals with severe depression did not respond to our survey. Although we were unable to examine the association of the severity of depression or the prevalence of anxiety only with online health information seeking in this study, these results indicate that internet users with mild depression or anxiety were likely to seek online health information and use SNSs. Examination of the association between depression severity and online health information seeking or SNS use is required in future studies. Besides, strategies using the internet appear to be effective, and reports of the use of information technologies in mental health care have increased in recent years [45]. However, several prior studies indicated a bidirectional relationship between social media use and depression or anxiety, and the risk of a downward depressive spiral effect related to SNSs [46,47]; whether this effect was beneficial or detrimental depends at least partly on the quality of social factors in the SNS environment [48]. Thus, future studies will be needed to identify the characteristics of individuals with depression or anxiety who can benefit from the intervention using the internet.

This study revealed that several sociodemographic factors and health literacy were associated with online health information seeking and SNS use. Men, older age, lower education status, and being unmarried were associated with less online health information seeking, which is consistent with previous findings [11,12,16,49,50]. Men, older age, and lower education status were also associated with a lower likelihood of SNS use, which is also in accord with the findings of previous studies [10,13,49,51,52]. Websites and SNSs may be effective tools for health communication among internet users. It may be beneficial to encourage men, older adults, internet users with low education levels, and those with low health literacy to develop the ability to seek health information and to use SNSs. Furthermore, internet users who experienced worse health status were found to be more likely to use health information on the internet, consistent with previous studies [16,50]. This result may indicate that people are more likely to require health information when their health status is worse. Therefore, improving the online environment to enable easy access to websites providing reliable health information and improving the health literacy of individuals are important.

### Limitations

This study has several limitations. First, the response rate was low. Among the study respondents, the crude percentage of women and adults aged 50-69 years was higher than that among nonrespondents in terms of sex and age (Multimedia Appendix 1). This point should be considered when interpreting the results, even though we used a weighted analysis for the general population in Japan based on data from a national survey. In addition, we excluded respondents with missing data. In an additional analysis of our data including respondents with missing data, the proportion of older adults aged 70 or older and the proportion of individuals with chronic diseases were higher, and the proportion of the participants seeking health information on the internet and SNS was lower in respondents with missing data compared with those in the final sample for the analyses (Multimedia Appendix 2). If this is the case among nonrespondents, the prevalence of chronic diseases may have been underestimated, and the proportion of online health information seeking and SNS use may be overestimated in this study compared with the general Japanese population, even though we adjusted for nonrespondents. Moreover, our findings may not be directly generalizable to other countries because this study was conducted only in Japan. Second, this study was conducted during the COVID-19 pandemic. Although no studies have identified an effect of COVID-19 on online health information seeking, online health information seeking may have increased during the COVID-19 pandemic because approximately 90% of internet users accessed information about COVID-19 from the official government website [53]. Thus, the proportion of those seeking health information on the internet may have been overestimated in this study compared with data before or after the COVID-19 pandemic. Although a previous study showed a heavy reliance on social media during the COVID-19 pandemic [54], another study indicated limited use of SNS during the COVID-19 pandemic [55]. Future studies will be needed to examine the effect of the COVID-19 pandemic on SNS use in Japan. Despite these limitations, our findings have important implications for the management of chronic diseases and health promotion using the internet.

### Conclusions

For patients with cancer, strategies for promoting access to websites with reliable cancer-related information and access among patients with chronic lung diseases to YouTube videos providing reliable information may be beneficial for the management of these diseases. Moreover, it is important to improve the online environment to encourage men, older adults, internet users with low education levels, and those with low health literacy to access online health information.

## Appendix

Multimedia Appendix 1 Crude percentages of respondents and nonrespondents in this study.

Multimedia Appendix 2 Weighted percentages of final sample for analyses and respondents with missing data.

## Abbreviations

AOR: adjusted odds ratio
CVA: cerebrovascular diseases including stroke
HINTS: Health Information National Trends Survey
QOL: quality of life
SNS: social networking site
WHO: World Health Organization

## References

[1] Mitsutake S, Ishizaki T, Teramoto C, Shimizu S, Ito H (2019). Patterns of co-occurrence of chronic disease among older adults in Tokyo, Japan. Prev Chronic Dis.

[2] Barnett K, Mercer SW, Norbury M, Watt G, Wyke S, Guthrie B (2012). Epidemiology of multimorbidity and implications for health care, research, and medical education: a cross-sectional study. The Lancet.

[3] National Cancer Institute (NCI) (2021). HINTS 5, Cycle 4 (2020) Data Set. HINTS.

[4] (2021). Ministry of Public Management, Home Affairs, Posts and Telecommunications.

[5] König R, Seifert A, Doh M (2018). Internet use among older Europeans: an analysis based on SHARE data. Univ Access Inf Soc.

[6] Madrigal L, Escoffery C (2019). Electronic health behaviors among US adults with chronic disease: cross-sectional survey. J Med Internet Res.

[7] Tan SS, Goonawardene N (2017). Internet Health Information Seeking and the Patient-Physician Relationship: A Systematic Review. J Med Internet Res.

[8] Luo A, Qin L, Yuan Y, Yang Z, Liu F, Huang P, Xie W (2022). The Effect of Online Health Information Seeking on Physician-Patient Relationships: Systematic Review. J Med Internet Res.

[9] Qin L, Zhang X, Wu A, Miser JS, Liu Y, Hsu JC, Shia B, Ye L (2021). Association Between Social Media Use and Cancer Screening Awareness and Behavior for People Without a Cancer Diagnosis: Matched Cohort Study. J Med Internet Res.

[10] Huo J, Desai R, Hong Y, Turner K, Mainous AG, Bian J (2019). Use of Social Media in Health Communication: Findings From the Health Information National Trends Survey 2013, 2014, and 2017. Cancer Control.

[11] Din HN, McDaniels-Davidson C, Nodora J, Madanat H (2019). Profiles of a Health Information-Seeking Population and the Current Digital Divide: Cross-Sectional Analysis of the 2015-2016 California Health Interview Survey. J Med Internet Res.

[12] Kontos E, Blake KD, Chou WS, Prestin A (2014). Predictors of eHealth usage: insights on the digital divide from the Health Information National Trends Survey 2012. J Med Internet Res.

[13] Langford A, Loeb S (2019). Perceived Patient-Provider Communication Quality and Sociodemographic Factors Associated With Watching Health-Related Videos on YouTube: A Cross-Sectional Analysis. J Med Internet Res.

[14] Xiong Z, Zhang L, Li Z, Xu W, Zhang Y, Ye T (2021). Frequency of Online Health Information Seeking and Types of Information Sought Among the General Chinese Population: Cross-sectional Study. J Med Internet Res.

[15] Nelson DE, Kreps GL, Hesse BW, Croyle RT, Willis G, Arora NK, Rimer BK, Viswanath KV, Weinstein N, Alden S (2004). The Health Information National Trends Survey (HINTS): development, design, and dissemination. J Health Commun.

[16] Finney Rutten LJ, Blake KD, Skolnick VG, Davis T, Moser RP, Hesse BW (2020). Data Resource Profile: The National Cancer Institute's Health Information National Trends Survey (HINTS). Int J Epidemiol.

[17] World Health Organization (WHO) (2021). Sample size calculator. World Health Organization (WHO).

[18] Otsuki A, Saito J, Yaguchi‐Saito A, Odawara M, Fujimori M, Hayakawa M, Katanoda K, Matsuda T, Matsuoka YJ, Takahashi H, Takahashi M, Inoue M, Yoshimi I, Kreps GL, Uchitomi Y, Shimazu T (2022). A nationally representative cross‐sectional survey on health information access for consumers in Japan: A protocol for the INFORM Study. World Med & Health Policy.

[19] Monthly Monetary and Financial Statistics (MEI): Exchange rates (USD monthly averages). Organisation for Economic Co-operation and Development (OECD).

[20] Ishikawa H, Nomura K, Sato M, Yano E (2008). Developing a measure of communicative and critical health literacy: a pilot study of Japanese office workers. Health Promot Int.

[21] National Cancer Institute (2020). HINTS 5: Cycle 4 Methodology Report. HINTS.

[22] Wolter K (2007). Introduction to Variance Estimation. 2nd ed.

[23] Sedrak MS, Soto-Perez-De-Celis E, Nelson RA, Liu J, Waring ME, Lane DS, Paskett ED, Chlebowski RT (2020). Online Health Information-Seeking Among Older Women With Chronic Illness: Analysis of the Women's Health Initiative. J Med Internet Res.

[24] Dee E, Muralidhar V, Butler S, Yu Z, Sha S, Mahal B, Nguyen Paul L, Sanford Nina N (2020). General and Health-Related Internet Use Among Cancer Survivors in the United States: A 2013-2018 Cross-Sectional Analysis. J Natl Compr Canc Netw.

[25] Mattsson S, Olsson EMG, Johansson B, Carlsson M (2017). Health-Related Internet Use in People With Cancer: Results From a Cross-Sectional Study in Two Outpatient Clinics in Sweden. J Med Internet Res.

[26] Finney Rutten Lila J, Agunwamba AA, Wilson P, Chawla N, Vieux S, Blanch-Hartigan D, Arora NK, Blake K, Hesse BW (2016). Cancer-Related Information Seeking Among Cancer Survivors: Trends Over a Decade (2003-2013). J Cancer Educ.

[27] Husson O, Mols F, van de Poll-Franse L (2011). The relation between information provision and health-related quality of life, anxiety and depression among cancer survivors: a systematic review. Ann Oncol.

[28] Grewal P, Alagaratnam S (2013). The quality and readability of colorectal cancer information on the internet. Int J Surg.

[29] Fefer M, Lamb CC, Shen AH, Clardy P, Muralidhar V, Devlin PM, Dee EC (2020). Multilingual Analysis of the Quality and Readability of Online Health Information on the Adverse Effects of Breast Cancer Treatments. JAMA Surg.

[30] Charow R, Snow M, Fathima S, Giuliani ME, McEwan K, Winegust J, Papadakos J (2019). Evaluation of the scope, quality, and health literacy demand of Internet-based anal cancer information. J Med Libr Assoc.

[31] Choudhery S, Xi Y, Chen H, Aboul-Fettouh N, Goldenmerry Y, Ho C, Viroslav H, Zhang C, Goudreau S (2020). Readability and Quality of Online Patient Education Material on Websites of Breast Imaging Centers. J Am Coll Radiol.

[32] Chen Y, Li C, Liang J, Tsai C (2018). Health Information Obtained From the Internet and Changes in Medical Decision Making: Questionnaire Development and Cross-Sectional Survey. J Med Internet Res.

[33] Norman CD, Skinner HA (2006). eHealth Literacy: Essential Skills for Consumer Health in a Networked World. J Med Internet Res.

[34] Xu RH, Zhou L, Wong EL, Wang D (2021). The Association Between Patients' eHealth Literacy and Satisfaction With Shared Decision-making and Well-being: Multicenter Cross-sectional Study. J Med Internet Res.

[35] Mitsutake S, Shibata A, Ishii K, Miyawaki R, Oka K (2020). Associations of eHealth Literacy with Obtaining Knowledge about Colorectal Cancer among Internet Users Accessing a Reputable Cancer Website: Internet-Based Survey Study. Int J Environ Res Public Health.

[36] De Main AS, Xie B, Shiroma K, Yeh T, Davis N, Han X (2022). Assessing the Effects of eHealth Tutorials on Older Adults' eHealth Literacy. J Appl Gerontol.

[37] Schrijver J, Lenferink A, Brusse-Keizer M, Zwerink M, van der Valk Paul Dlpm, van der Palen Job, Effing Tanja W (2022). Self-management interventions for people with chronic obstructive pulmonary disease. Cochrane Database Syst Rev.

[38] Santino T, Chaves G, Freitas D, Fregonezi G, Mendonça Karla Mpp (2020). Breathing exercises for adults with asthma. Cochrane Database Syst Rev.

[39] Stellefson M, Chaney B, Ochipa K, Chaney D, Haider Z, Hanik B, Chavarria E, Bernhardt JM (2014). YouTube as a source of chronic obstructive pulmonary disease patient education: a social media content analysis. Chron Respir Dis.

[40] Diers CS, Remvig C, Meteran H, Thomsen SF, Sigsgaard T, Høj Simon, Meteran H (2022). The usefulness of YouTube videos as a source of information in asthma. J Asthma.

[41] Japan Asthma Society Video of how to use the inhaled drug. Japan Asthma Society.

[42] Berle D, Starcevic V, Khazaal Y, Viswasam K, Hede V, McMullan RD (2020). Relationships between online health information seeking and psychopathology. Gen Hosp Psychiatry.

[43] Khazaal Y, Chatton A, Rochat L, Hede V, Viswasam K, Penzenstadler L, Berle D, Starcevic V (2021). Compulsive Health-Related Internet Use and Cyberchondria. Eur Addict Res.

[44] Park S, Kim D (2020). The Centrality of Depression and Anxiety Symptoms in Major Depressive Disorder Determined Using a Network Analysis. J Affect Disord.

[45] Timakum T, Xie Q, Song M (2022). Analysis of E-mental health research: mapping the relationship between information technology and mental healthcare. BMC Psychiatry.

[46] Lopes LS, Valentini JP, Monteiro TH, Costacurta MCDF, Soares LON, Telfar-Barnard L, Nunes PV (2022). Problematic Social Media Use and Its Relationship with Depression or Anxiety: A Systematic Review. Cyberpsychol Behav Soc Netw.

[47] Takahashi Y, Uchida C, Miyaki K, Sakai M, Shimbo T, Nakayama T (2009). Potential benefits and harms of a peer support social network service on the internet for people with depressive tendencies: qualitative content analysis and social network analysis. J Med Internet Res.

[48] Seabrook EM, Kern ML, Rickard NS (2016). Social Networking Sites, Depression, and Anxiety: A Systematic Review. JMIR Ment Health.

[49] Calixte R, Rivera A, Oridota O, Beauchamp W, Camacho-Rivera M (2020). Social and Demographic Patterns of Health-Related Internet Use Among Adults in the United States: A Secondary Data Analysis of the Health Information National Trends Survey. Int J Environ Res Public Health.

[50] Lee HY, Jin SW, Henning-Smith C, Lee J, Lee J (2021). Role of Health Literacy in Health-Related Information-Seeking Behavior Online: Cross-sectional Study. J Med Internet Res.

[51] Tennant B, Stellefson M, Dodd V, Chaney B, Chaney D, Paige S, Alber J (2015). eHealth literacy and Web 2.0 health information seeking behaviors among baby boomers and older adults. J Med Internet Res.

[52] Chou WS, Gaysynsky A, Trivedi N, Vanderpool RC (2021). Using Social Media for Health: National Data from HINTS 2019. J Health Commun.

[53] Ali SH, Foreman J, Tozan Y, Capasso A, Jones AM, DiClemente RJ (2020). Trends and Predictors of COVID-19 Information Sources and Their Relationship With Knowledge and Beliefs Related to the Pandemic: Nationwide Cross-Sectional Study. JMIR Public Health Surveill.

[54] Neely S, Eldredge C, Sanders R (2021). Health Information Seeking Behaviors on Social Media During the COVID-19 Pandemic Among American Social Networking Site Users: Survey Study. J Med Internet Res.

[55] Skarpa PE, Garoufallou E (2021). Information seeking behavior and COVID-19 pandemic: A snapshot of young, middle aged and senior individuals in Greece. Int J Med Inform.

