# Supplementary material for: Chronic Diseases and Sociodemographic Characteristics Associated With Online Health Information Seeking and Using Social Networking Sites: Nationally Representative Cross-sectional Survey in Japan
Source: J Med Internet Res. 2023 Mar 2;25:e44741. doi: 10.2196/44741 (PMC10020913; doi:10.2196/44741)
Supplement: Multimedia Appendix 1 [file jmir_v25i1e44741_app1.docx]

Supplemental table 1. Crude percentages of respondents and non-respondent of the present study

| Characteristics | | Respondents  n=3605 | Non-respondents  n=6114 |
| --- | --- | --- | --- |
| Sex | Men | 45.6 | 51.2 |
|  | Women | 54.4 | 48.8 |
| Age groups | 20–29 | 8.2 | 11.5 |
|  | 30–39 | 11.2 | 13.1 |
|  | 40–49 | 17.9 | 18.5 |
|  | 50–59 | 18.1 | 15.6 |
|  | 60–69 | 19.4 | 13.4 |
|  | ≥ 70 | 25.1 | 27.8 |
