# Supplementary material for: Chronic Diseases and Sociodemographic Characteristics Associated With Online Health Information Seeking and Using Social Networking Sites: Nationally Representative Cross-sectional Survey in Japan
Source: J Med Internet Res. 2023 Mar 2;25:e44741. doi: 10.2196/44741 (PMC10020913; doi:10.2196/44741)
Supplement: Multimedia Appendix 2 [file jmir_v25i1e44741_app2.docx]

Supplemental table 2. Weighted percentages of final sample for analyses and respondents with missing data

| Characteristics | | Final sample n=2481^a^ | Respondents with missing data, n=321^a^ | P^b^ |
| --- | --- | --- | --- | --- |
| Sex | Men | 51.6 | 50.0 | 0.577 |
|  | Women | 48.4 | 50.0 |  |
| Age groups | 20–29 | 14.3 | 6.0 | <0.001 |
|  | 30–39 | 17.3 | 8.4 |  |
|  | 40–49 | 24.1 | 10.3 |  |
|  | 50–59 | 19.4 | 15.3 |  |
|  | 60–69 | 14.4 | 17.4 |  |
|  | ≥ 70 | 10.5 | 42.6 |  |
| Education status | ≤ High school graduate | 37.5 | 58.0 | <0.001 |
|  | Career college/junior college | 24.3 | 19.2 |  |
|  | ≥ College graduate | 38.2 | 21.0 |  |
|  | No answer or missing | 0.0 | 1.8 |  |
| Work | No | 25.8 | 46.4 | <0.001 |
|  | Yes | 74.2 | 51.1 |  |
|  | No answer or missing | 0.0 | 2.5 |  |
| Marital status | Not married | 32.3 | 34.5 | <0.001 |
|  | Married | 67.7 | 63.3 |  |
|  | No answer or missing | 0.0 | 0.2 |  |
| Household income (million yen) | < 2 | 5.6 | 18.1 | <0.001 |
|  | 2–< 4 | 21.6 | 27.5 |  |
|  | 4–< 6 | 23.8 | 18.6 |  |
|  | 6–< 8 | 19.5 | 12.1 |  |
|  | 8–< 10 | 12.4 | 9.7 |  |
|  | ≥ 10 | 14.9 | 9.3 |  |
|  | No answer or missing | 2.1 | 4.8 |  |
| Health literacy | 5–15 | 19.6 | 22.6 | <0.001 |
|  | 16–18 | 30.7 | 23.9 |  |
|  | 19–20 | 34.6 | 22.9 |  |
|  | ≥ 21 | 15.1 | 12.1 |  |
|  | No answer or missing | 0.0 | 18.5 |  |
| Self-reported health status | Excellent, very-good, good | 79.9 | 67.4 | <0.001 |
|  | Fair-poor | 20.1 | 28.3 |  |
|  | No answer or missing | 0.0 | 4.3 |  |
| **Chronic diseases** |  |  |  |  |
| Cancer | Yes | 7.2 | 18.2 | <0.001 |
| Diabetes or high blood sugar | Yes | 6.9 | 18.9 | <0.001 |
| High blood pressure or hypertension | Yes | 24.5 | 37.2 | <0.001 |
| Heart diseases | Yes | 3.9 | 7.5 | <0.001 |
| CVA | Yes | 1.0 | 1.8 | <0.001 |
| Lung diseases | Yes | 10.1 | 10.3 | <0.001 |
| Arthritis or rheumatism | Yes | 3.8 | 7.4 | <0.001 |
| Depression or anxiety disorder | Yes | 7.7 | 7.4 | <0.001 |
| **Dependent variables** | | | |  |
| Online health information seeking | Yes | 72.9 | 31.4 | <0.001 |
| **SNSs use** |  |  |  |  |
| Visiting a SNS | Yes | 55.3 | 15.9 | <0.001 |
| Sharing health information on SNS | Yes | 12.9 | 6.6 | <0.001 |
| Watching a health-related video on YouTube | Yes | 26.6 | 13.8 | <0.001 |
| Writing in an online diary or blog | Yes | 6.6 | 0.9 | <0.001 |

Abbreviations: CVA: Cerebrovascular diseases including stroke, SNS: Social networking site

a: Results were weighted to be representative of the population of Internet users in Japan.

b: Chi-square test.
